# Metastatic renal cell cancer treatments: An indirect comparison meta-analysis

**DOI:** 10.1186/1471-2407-9-34

**Published:** 2009-01-27

**Authors:** Edward J Mills, Beth Rachlis, Chris O'Regan, Lehana Thabane, Dan Perri

**Affiliations:** 1 Faculty of Health Sciences, Simon Fraser University, Vancouver, Canada; 2 Department of Clinical Epidemiology & Biostatistics, McMaster University, Hamilton, Canada; 3 Department of Public Health Sciences, University of Toronto, Toronto, Canada; 4 Department of Epidemiology, London School of Hygiene and Tropical Medicine, London, UK; 5 Centre for Evaluation of Medicines, St. Joseph's Healthcare, Hamilton, Canada; 6 Department of Medicine, McMaster University, Hamilton, Canada

## Abstract

**Background:**

Treatment for metastatic renal cell cancer (mRCC) has advanced dramatically with understanding of the pathogenesis of the disease. New treatment options may provide improved progression-free survival (PFS). We aimed to determine the relative effectiveness of new therapies in this field.

**Methods:**

We conducted comprehensive searches of 11 electronic databases from inception to April 2008. We included randomized trials (RCTs) that evaluated bevacizumab, sorafenib, and sunitinib. Two reviewers independently extracted data, in duplicate. Our primary outcome was investigator-assessed PFS. We performed random-effects meta-analysis with a mixed treatment comparison analysis.

**Results:**

We included 3 bevacizumab (2 of bevacizumab plus interferon-a [IFN-a]), 2 sorafenib, 1 sunitinib, and 1 temsirolimus trials (total n = 3,957). All interventions offer advantages for PFS. Using indirect comparisons with interferon-α as the common comparator, we found that sunitinib was superior to both sorafenib (HR 0.58, 95% CI, 0.38–0.86, P = < 0.001) and bevacizumab + IFN-a (HR 0.75, 95% CI, 0.60–0.93, P = 0.001). Sorafenib was not statistically different from bevacizumab +IFN-a in this same indirect comparison analysis (HR 0.77, 95% CI, 0.52–1.13, P = 0.23). Using placebo as the similar comparator, we were unable to display a significant difference between sorafenib and bevacizumab alone (HR 0.81, 95% CI, 0.58–1.12, P = 0.23). Temsirolimus provided significant PFS in patients with poor prognosis (HR 0.69, 95% CI, 0.57–0.85).

**Conclusion:**

New interventions for mRCC offer a favourable PFS for mRCC compared to interferon-α and placebo.

## Background

Renal cell carcinoma results in an estimated 54,000 new cases and 13,000 deaths each year in the United States [1]. Approximately 30% of patients present with metastatic disease (mRCC), often associated with poor prognosis and subsequent 5-year survival rate [2,3] Until recently, treatment was limited to cytokine therapies (interleukin-2 [IL-2] and interferon-alfa [IFN-α ]) that produce modest response rates (< 20%) and substantial toxicities, although occasional complete responses have been reported [4]. Findings from randomized trials have shown that treatment with cytokine therapy results in an overall median survival of 13 months (range 6–28) [5].

As a greater understanding of the molecular mechanisms involved in the pathogenesis of metastatic renal cell carcinoma (mRCC) develops, more advanced treatment options are emerging [4,6]. Clear-cell carcinoma histology in particular, is associated with the von Hippel-Lindau (VHL) tumour suppressor gene. Loss of this gene function results in an over expression of several hypoxia-responsive proteins, including the vascular endothelial growth factor (VEGF) and platelet derived growth factor (PDGF), capable of promoting tumour growth and angiogenesis [7]. Newer antiangiogenesis-targeting agents are targeting these factors and have recently provided more promising treatment options. New VEGF targeted regimens (bevacizumab, sorafenib, and sunitinib) and temsirolimus (CCI-779), an inhibitor of mammalian target of rapamycin (mTOR) kinase, in particular, have now been evaluated, compared to interferon as a first-line treatment[6], although they have not been directly evaluated in head-to-head trials. Sunitinib and sorafenib have shown positive PFS endpoints when compared to IFN-α or placebo, in phase III trials [8,9]. Similarly, phase III trials of bevacizumab plus interferon have shown superiority in PFS, response, and tolerability compared with interferon alone [4]. Temsirolimus has demonstrated positive PFS in patients with a poor prognosis [10].

In a commentary in *The Lancet*, Motzer and Basch (2007) highlight that important information on the clinical setting, nature of the treatment program, and survival outcomes is necessary to inform the management of patients as well as future trial designs [6]. Other considerations such as cost, patient experience, and adverse events also have an important place in the decision-making process and have complicated the delivery of these new interventional drugs in some settings [11]. Thus, to determine optimal treatment in a rapidly advancing era of targeted therapy [6], we conducted a meta-analyses of all randomized controlled trials examining either bevacizumab, sorafenib, sunitinib or temsirolimus for the treatment of mRCC. In the absence of head-to-head evaluations, we applied an adjusted indirect comparison analysis [12].

## Methods

### Eligibility criteria

We included any randomized clinical trial evaluating the therapeutic efficacy of VEGF inhibitors bevacizumab, sorafenib, or sunitinib, and the mTOR inhibitor temsirolimus for the treatment of mRCC. Studies had to evaluate one of the study drugs with a control intervention. We included trials involving patients of any age, sex, or mRCC stage. We included trials using the aforementioned study drugs as either sole treatment and as adjunct treatment. We excluded pharmacokinetic studies, non-randomized evaluations, early results presentations (when later results were available), and animal/laboratory studies.

### Search Strategy

In consultation with an information specialist (PR), 2 searchers (EM, BR) developed search strategies. First, we identified key terms of the study drugs with their trade names. We then identified the various permutations of mRCC listings in databases. The searches combined these terms with the cancer MeSH heading "neoplasms" from January 2008 to April 8th 2008. We searched independently, in duplicate (EM, BR), the following 11 databases (from inception to April 8th 2008): AMED, CinAhl, Cochrane Library (inception to issue 1, 2008), Embase, and MedLine via PubMed. We sought unpublished studies through "clinicaltrials.gov", the UK National Research Register and conference abstracts available from the American Society of Clinical Oncology (ASCO) website, which includes 18 annual conferences of ASCO and Genitourinary Cancers Symposium, Gastrointestinal Cancers Symposium, Breast Cancer Symposium, Prostate Cancer Symposiums dating to before 2004. We supplemented this search by reviewing the bibliographies of key papers.

### Study selection and Data extraction

Two reviewers (BR and EM) independently extracted data and appraised both quality and content. We conducted data extraction using a standardized form (available from corresponding author upon request). Initially, abstracts were screened to exclude obviously ineligible reports, and complete primary reports were reviewed for all remaining studies. We classified trials and abstracts according to study drug under investigation (i.e., bevacizumab, sorafenib, sunitinib, and temsirolimus) and obtained information on patient characteristics, study design and therapy duration. Study design items included methods of sequence generation, allocation concealment, complete description of who was blinded, use of intention-to-treat analysis and whether the trial was stopped prior to the planned duration, all methodological features capable of impacting effect sizes [13,14] Our primary endpoint was progression-free survival (PFS) as defined by investigators, or where unavailable, by independent reviewers. We were also interested in overall survival, median duration of treatment and duration of treatment response, and the overall response rate. Adverse events and details on deaths were recorded when available. We entered the data into an electronic database such that duplicate entries existed for each study; when two entries did not match, we reached consensus through discussion and 3rd party arbitration (CO). To obtain full information regarding conference abstracts, we attempted contact with the study authors for full information through email and telephone communication.

### Data analysis

In order to assess inter-rater reliability regarding eligibility, we calculated the *Phi *statistic (φ), which provides a measure of inter-observer agreement independent of chance [15] We calculated the hazard ratio (HR) and appropriate 95% confidence intervals (CIs) of PFS according to the HR reported in the original studies, communication with the authors, or through our own recalculations. We used the last available report of each study outcomes. We combined studies when more than one trial of the four target drugs existed and were clinically sensible. We pooled studies for each drug using the DerSimonian-Laird random effects model [16], which recognizes and anchors studies as a sample of all potential studies, and incorporates an additional between-study component to the estimate of variability [17]. In the absence of head-to-head evaluations, we conducted indirect comparisons of the interventions versus similar comparators across similar patient groups using methods described by Bucher et al [12]. This method maintains the randomization from each trial and compares the summary estimates of pooled interventions with CIs. We used forest plots to display the trial outcomes. We present the geometric distribution of the indirect comparisons to display which comparisons were evaluated. We used SAS (Cary, NC, version 9.0) for all analysis.

## Results

**Figure 1:**
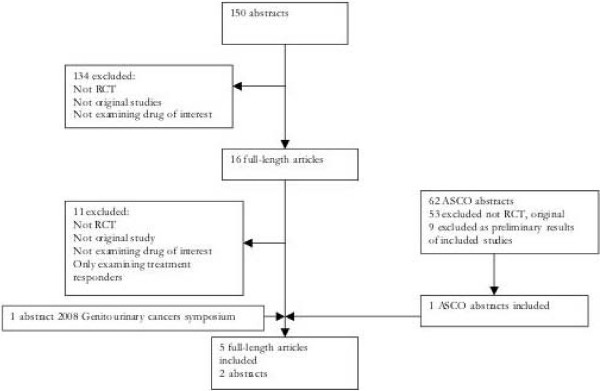
**Flow diagram of included studies**.

Our primary literature search identified 150 full-length articles. There was excellent agreement between reviewers on narrowing this to 16 full-text articles review (φ = 0.87). Of these [11], were excluded as they were not original studies, were not randomized controlled trials, did not examine at least one of the selected drug therapies (i.e., bevacizumab, sunitinib) or in one case, only examined outcomes in treatment responders. The remaining 5 reports were included. While numerous abstracts from relevant conferences and annual meetings were reviewed, only 2 were included [18,19] as they reported original studies. One of these [18] was subsequently published in full during the review stage of this manuscript and checked for accuracy [20] Additionally, 9 abstracts examined reported on the preliminary findings of 5 included studies [21-29] and were subsequently excluded. Figure 1 displays the flow-diagram of the 7 included studies totalling 3,957 patients.

In total, 2 full-length studies [4,30] and 1 abstract [18] examined bevacizumab; 1 full-length study [31] and 1 abstract [19] examined sorafenib; 1 full-length report [8] presented follow-up data examining sunitinib for the treatment of mRCC; and 1full-length report examined temsirolimus [10]. Interferon-α was the most common comparator in 5 included studies [4,18,19,8,10] while placebos were used in 2 studies [23,9] Study and patient population characteristics for each included trial are displayed in Additional File 1 [See Additional File 1].

While all included studies were randomized controlled trials, among the 5 full-length articles, only 1 detailed the sequence generation process [4] Only this same study adequately described allocation concealment. Descriptions of who was blinded were available in 3 studies [4,8,31]. All 5 full-length reports employed an intent-to-treat design in their statistical analyses [4,8,30,31,10]. Finally, all 5 full length reports described adequate safety monitoring, with all five employing the use of a data and safety monitoring board (DSMB) [4,8,30,31,10]. Four trials employed their stopping rules [8,9,30,19]. Treatment outcomes by study are displayed in Additional File 2 [See Additional File 2].

### Meta-analysis of PFS

**Figure 2:**
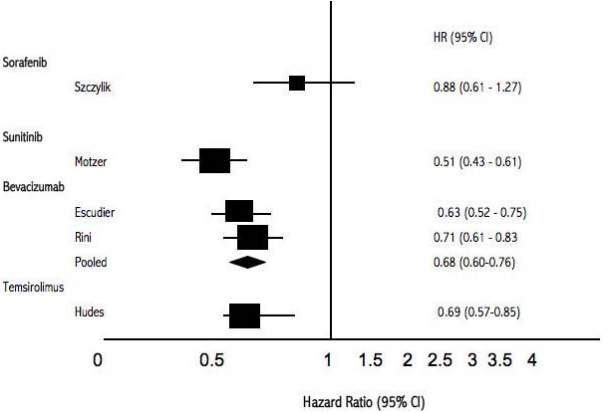
**Forest plot Hazard Rations (95% CI) of intervention versus IFN-α**.

**Figure 3:**
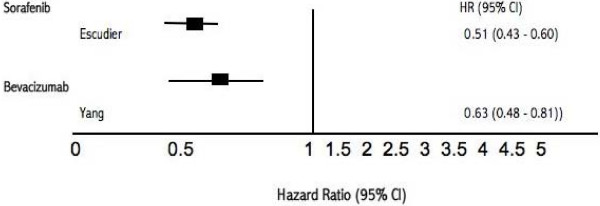
**Forest plot Hazard Rations (95% CI) of intervention versus placebo**.

Five trials examined the new therapeutic interventions compared to IFN-α for PFS. One trial evaluated temsirolimus in patients with a poor prognosis. We pooled 2 trials that evaluated bevacizumab plus interferon versus interferon alone (total n = 1,381) and found a pooled HR of 0.68 (95% CI, 0.60–0.76, P = 0.001). We did not pool the remaining studies as they had differing interventions. Figure 2 displays the treatment versus IFN-α PFS outcomes graphically and figure 3 displays the interventions versus placebo. We did not pool study outcomes for survival.

### Indirect comparisons of interventions versus IFN-α and placebo for PFS

**Figure 4:**
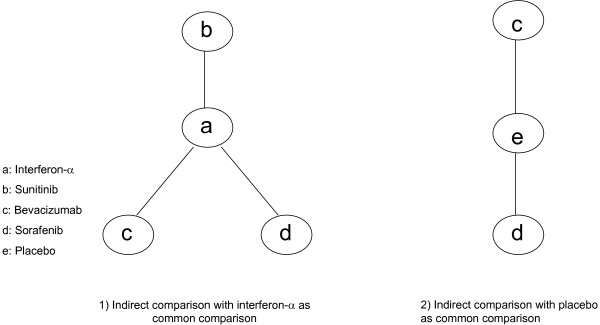
**Geometric distribution of adjusted indirect comparisons**.

Figure 4 displays the geometric distribution of the indirect comparisons [32]. As the study evaluating temsirolimus [10] included patients that were clinically different than the other trials we did not include the temsirolimus population in our indirect comparison evaluation. When we evaluated the treatment effects of sunitinib versus sorafenib, we identify superiority of sunitinib (HR 0.58, 95% CI, 0.38–0.86, P = < 0.001) [8,19]. When we evaluate the effect of sunitinib [8] versus bevacizumab plus interferon[4,18], we identify the superiority of sunitinib (HR 0.75, 95% CI, 0.60–0.93, P = 0.001). When we evaluated the effects of sorafenib [19] versus bevacizumab plus interferon [4,18], we were unable to demonstrate significant differences (HR 0.77, 95% CI, 0.52–1.13, P = 0.21).

**Figure 5:**
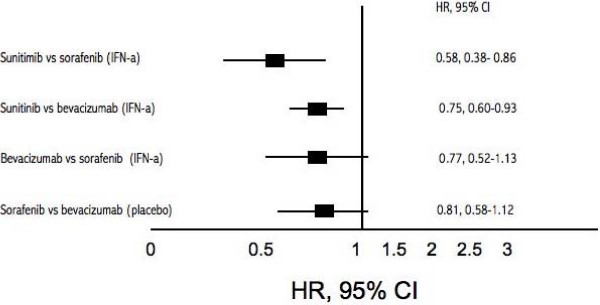
**Forest plot Hazard Rations (95% CI) of adjusted indirect comparisons of interventions versus IFN-α or placebo**.

We further evaluated an indirect comparison between sorafenib and bevacizumab alone, with placebo as the similar comparator in previously treated patients through the indirect comparison of two studies [9,23] We were unable to display a significant difference (HR 0.81, 95% CI, 0.58–1.12. P = 0.23). Figure 5 displays the comparisons graphically.

## Discussion

Our study should be of interest to patients and clinicians alike. Until only recently, mRCC patients had few treatment options beyond cytokine therapies. Our study demonstrates consistent PFS benefits with the new targeted therapies. Insights into the histology of RCC appear to now result in considerably improved PFS and response rates. Our study represents a first step at determining the relative effectiveness of each new intervention.

We sought out all randomized controlled trials of targeted drugs for mRCC. In the end, five papers and two abstracts met our inclusion criteria. Analysis of the populations from those studies revealed there to be three distinct groups of patients: treatment naïve patients of favorable to intermediate risk, refractory patients of favorable to intermediate risk, and refractory patients of intermediate to poor risk. As we hoped to measure the overall treatment effect of targeted therapy, we included both the treatment naïve and refractory patients of favorable to intermediate risk. While the refractory group were likely to be enrolled later after their time of diagnosis, and hence at risk for shorter PFS by definition, we felt that PFS was still a reasonable outcome measure based on their favorable (and similar) prognostic risk. In a pooled analysis this would likely bias the results in a negative direction that would make a statistically positive result more likely to be true. For patients with a poorer prognosis, PFS (our *a priori *primary endpoint) is likely shorter whether or not previous treatment has been tried. Interestingly, as can be seen in Additional Files 1 and 2 [See additional Files 1 and 2], median duration of treatment and hazard ratios for PFS and investigator assessed response rate were comparable between the poor-risk patients and favorable-risk ones.

Establishing prognosis of mRCC is difficult as there are a variety of patient, tumour, and biochemical factors that have been shown to influence prognosis [33-35]. Some of these include, among other variables, prior nephrectomy, tissue pathology, tumour stage (nodes and number of metastatic sites), serum calcium, alkaline phosphatase, and hemoglobin values, and scores of performance such as Eastern Co-operative Oncology Group (ECOG) performance status or Karnofsky score. We accepted individual study authors' assessment of prognosis into favorable-risk, intermediate, and poor-risk patients. While some criteria varied between papers, measures of ECOG or Karnofsky scores were also used by authors and we accepted risk stratification based upon them. From Additional Files 1 and 2 [See Additional Files 1 and 2], however, the PFS, and clinician determined response to therapy was similar between the other groups included in our statistical models. While they are not perfectly matched groups from a prognostic point of view, we felt they are similar enough for inclusion into our analysis. Further limitations of included trials based on more specific prognostic factors would have made any analysis impossible given the variety of different prognostic scores available for metastatic renal cell carcinoma.

There are several important strengths and limitations to consider when interpreting our analysis. Strengths include our extensive searching and successful contact with authors for clarifications and in one case, access to individual patient data. Our data analysis approach used a strategy that permits strong inferences about the relative effectiveness of interventions in the absence of head-to-head direct evidence. While head-to-head trials remain the highest level of evidence of therapeutic effectiveness, the method we employed has demonstrated comparatively consistent findings between this indirect method and head-to-head trials [36,37]. Limitations of our review include the absence of head-to-head trials, the potential that other trials exist that we were unable to identify, and the small number of included studies, thus negating the possibility of exploring possible sub-group analyses and exploring heterogeneity among study populations. Indeed, such analyses can only be undertaken when there are a large number of included studies.

Our method of analysis employed the adjusted indirect comparisons meta-analysis method. This method may be unfamiliar to some readers. Strengths of this method are that it borrows strength from each trial and maintains the benefits of randomization. Clinical, as well as statistical, rationale is required for combining treatments. Head-to-head (direct comparisons) trials remain the highest level of evidence of therapeutic effectiveness and in our review the only head-to-head trials compared these new therapies versus cytokine therapy, IFN-α. While our comparative data is hypothesis generating, it highlights the need for appropriate new clinical trials that examine the direct relative effectiveness of these interventions and also examine the combined effectiveness of these interventions.

There has been a recent public outcry over access to these mRCC interventions in the UK. The UK National Institute for Clinical Excellence (NICE) has issued provisional guidance not to recommend any of the drugs on the National Health Service due to their cost [11]. Our review finds that the clinical benefits of these interventions are clear. However, NICE evaluated the cost of the drugs per quality adjusted life year (QALY) and found all four treatments exceeding the Institute's £30,000 threshold (bevacizumab £171,301, sorafenib tosylate £102,498, sunitinib £71,462, temsirolimus £94,385). This troubling finding indicates two important public health challenges: 1) drugs need to be available at reduced costs; and 2) thresholds for cost-effectiveness need to be adjusted according to relative clinical efficacy [38]. In May 2009, NICE will issue final guidance that will take into account new supplementary criteria to be applied in drugs used at 'End of Life', and because of this it is expected that one or more of the reviewed drugs will be recommended. It is difficult to envision our own clinical decision-making, if cost alone were the deciding factor.

## Conclusion

The findings of our study raise intriguing questions about the future of treatment options for mRCC. Sunitinib, bevacizumab, and temsirolimus all offer improved PFS compared to IFN-α. The current availability of these active agents allows new treatment options and strategies to extend PFS, but in some settings are too expensive for public availability. The optimal treatment strategy in mRCC is likely to be defined in future randomized trials that should examine combination therapies of these active agents.

## Competing interests

Over the past 5 years Edward Mills has consulted to Pfizer Ltd., Mac Reviews Inc., World Health Organization, GSK, and Boehringher Ingelheim: all on methodological issues. Chris O'Regan has been employed by Pfizer Ltd. Lehana Thabane consults with GSK on statistical issues. Beth Rachlis has no conflict of interest to declare. Dan Perri has no conflict of interest to declare.

## Authors' contributions

EM, CO, BR, and DP conceived the study. EM, CO, BR, LT, and DP acquired the data. EM, CO, BR, LT, and DP analyzed the data. EM, CO, BR, LT, and DP wrote and approved the final manuscript.

## Pre-publication history

The pre-publication history for this paper can be accessed here:

http://www.biomedcentral.com/1471-2407/9/34/prepub

## Supplementary Material

Additional File 1 **Study and patient population characteristics of included studies.** Included studies Click here for file

Additional File 2 **Summary of efficacy outcomes for each included study.** Outcomes Click here for file
