# Supplementary material for: Metastatic renal cell cancer treatments: An indirect comparison meta-analysis
Source: BMC Cancer. 2009 Jan 27;9:34. doi: 10.1186/1471-2407-9-34 (PMC2637892; doi:10.1186/1471-2407-9-34)
Supplement: Additional File 1 — Study and patient population characteristics of included studies. Included studies [file 1471-2407-9-34-S1.doc]

Additional File 1: Study and patient population characteristics of included studies

| **Author/year** | **Sample size (n)** | | | **Intervention** | **Control** | **Patient population** | **Median age** | **Female** | **Study Design** | **Median duration on treatment (mo)** | |
| --- | --- | --- | --- | --- | --- | --- | --- | --- | --- | --- | --- |
|  | **Total** | **Active** | **Control** |  |  |  |  |  |  | **Active** | **Control** |
| ***Bevacizumab*** |  |  |  |  |  |  |  |  |  |  |  |
| Escudier, 2007 | 649 | 327 | 322 | BEV 10 mg/kg every two weeks + interferon, 9MIU 3 times/wk in repeated cycles | Interferon | Favourable and intermediate risk, previous nephroctomy, Karnofsky performance >70%, no prior treatment | 61 active, 60 control | 29.7% | Phase III | 9.7 | 5.1 |
| Rini, 2008 | 732 |  |  | BEV 10mg/kg every two weeks + interferon, 9MIU 3 times/wk | Interferon | No previous treatment, clear cell RCC |  |  | Phase III |  |  |
| Yang, 2003 | 108 | 76 | 40 | BEV low dose: 3mg/kg or high dose: 10mg/kg of body weight given every 2 weeks | Placebo | Poor risk, ECOG ≤ 2, previous therapy with IL-2 | 53 high-dose, 54 low-dose, 53 placebo | 28% | Phase II |  |  |
| ***Sorafenib*** |  |  |  |  |  |  |  |  |  |  |  |
| Escudier, 2007 | 903 | 451 | 452 | SOR 400 mg twice daily | Placebo | Intermediate and favorable-risk status, ECOG of 0 or 1, previous treatment, clear cell | 58 active 59 placebo | 27.5% | Phase III | 23 wk | 12 wk |
| Szczylik, 2007 | 189 | 97 | 92 | SOR 400 mg twice daily | Interferon | Previously untreated patients, clear cell carcinoma |  |  | Phase II |  |  |
| ***Sunitinib*** |  |  |  |  |  |  |  |  |  |  |  |
| Motzer, 2007 | 750 | 375 | 375 | SUN 50 mg orally daily for 4 wk then 2 wk off in repeated cycles | Interferon | Favourable or intermediate  risk, clear cell, no prior systemic treatment, ECOG of 0 or 1 |  |  | Phase III | 11 | 4 |
| ***Temsirolimus*** |  |  |  |  |  |  |  |  |  |  |  |
| Hudes, 2007 | 626 | 419 | 207 | TEM 25mg weekly infusion, repeated cycles | Interferon | Previously untreated.  Modified poor risk, Karnofsky performance score: >60, no previous therapy | 59 | 31% | Phase III | 17 wk | 8 wk |

BEV=Bevacizumab; SOR= Sorafenib; SUN=Sunitinib; TEM=Temsirolimus
