# Supplementary material for: Metastatic renal cell cancer treatments: An indirect comparison meta-analysis
Source: BMC Cancer. 2009 Jan 27;9:34. doi: 10.1186/1471-2407-9-34 (PMC2637892; doi:10.1186/1471-2407-9-34)
Supplement: Additional File 2 — Summary of efficacy outcomes for each included study. Outcomes [file 1471-2407-9-34-S2.doc]

Additional file 2: Summary of efficacy outcomes for each included study

| **Author, Year** | **Intervention** | **Control** | **Independent Assessed PFS** | **Investigator Assessed PFS** | **Overall survival** | **Independent Assessed Response rate** | **Investigator**  **Assessed Response Rate** | **Deaths** | |
| --- | --- | --- | --- | --- | --- | --- | --- | --- | --- |
|  |  |  | **HR**  **95% CI** | **HR**  **95% CI** | **HR**  **95% CI** | **RR**  **95% CI** | **RR**  **95% CI** | **Active** | **Control** |
| ***Bevacizumab*** |  |  |  |  |  |  |  |  |  |
| Escudier, 2007 | BEV+ interferon | Interferon |  | 0.63  (0.52-0.75) | 0.79  (0.62-1.02) |  | 1.80  (1.53-2.12) | 114 | 137 |
| Rini, 2008 | BEV+ interferon | Interferon |  | 0.71  (0.61-0.83) |  |  | 25.5% vs. 13.1%  (p <0.001) |  |  |
| Yang, 2003* | BEV | Placebo |  | 0.63  (0.48-0.81) |  |  | All responses (n= 4) in high-dose group only |  |  |
| ***Sorafenib*** |  |  |  |  |  |  |  |  |  |
| Escudier, 2007 | SOR | Placebo | 0.44  (0.35-0.55) | 0.51  (0.43-0.60) | 0.72  (0.54-0.94) |  | 5.51  (2.62-11.58) | 97 | 123 |
| Szcylik, 2007 | SOR | Interferon |  | 0.88  (0.61-1.27) |  |  | 0.59  (0.20-173) |  |  |
| ***Sunitinib*** |  |  |  |  |  |  |  |  |  |
| Motzer, 2007 | SUN | Interferon | 0.54  (0.44-0.66) | 0.51  (0.43-0.61) |  | 4.64  (3.20-6.73) | 3.86  (2.87-5.18) |  |  |
| ***Temsirolimus*** |  |  |  |  |  |  |  |  |  |
| Hudes, 2007 | TEM | Interferon |  | 0.69  (0.57-0.85) | 0.73  (0.58-0.92) |  | 1.78  (0.84-3.77) |  |  |

BEV = Bevacizumab; SOR = Sorafenib; SUN = Sunitinib; TEM = Temsirolimus ; PFS = Progression Free Survival; HR=Hazard Ratio; RR= Response rate;

CI = Confidence Interval. * data provided by author.
